# Effects of Endurance Exercise Intensities on Autonomic and Metabolic Controls in Children with Obesity: A Feasibility Study Employing Online Exercise Training

**DOI:** 10.3390/nu15041054

**Published:** 2023-02-20

**Authors:** Valeria Calcaterra, Giuseppina Bernardelli, Mara Malacarne, Matteo Vandoni, Savina Mannarino, Vittoria Carnevale Pellino, Cristiana Larizza, Massimo Pagani, Gianvincenzo Zuccotti, Daniela Lucini

**Affiliations:** 1 Pediatric Unit, Pediatric Department, Buzzi Children’s Hospital, 20154 Milan, Italy; 2 Department of Internal Medicine, University of Pavia, 27100 Pavia, Italy; 3 Exercise Medicine Unit, Istituto Auxologico Italiano, IRCCS, 20135 Milan, Italy; 4 DISCCO Department, University of Milan, 20122 Milan, Italy; 5 BIOMETRA Department, University of Milan, 20129 Milan, Italy; 6 Laboratory of Adapted Motor Activity (LAMA), Department of Public Health, Experimental Medicine and Forensic Science, University of Pavia, 27100 Pavia, Italy; 7 Pediatric Cardiology Unit, Pediatric Department, Buzzi Children’s Hospital, 20154 Milan, Italy; 8 Department of Industrial Engineering, University of Rome Tor Vergata, 00133 Rome, Italy; 9 Department of Electrical, Computer and Biomedical Engineering, University of Pavia, 27100 Pavia, Italy; 10 Department of Biomedical and Clinical Science, University of Milano, 20157 Milan, Italy

**Keywords:** exercise intensity, training, autonomic nervous system, heart rate variability, metabolic control, children, online exercise training, obesity

## Abstract

Exercise is one of the major determinants of a healthy lifestyle, which is particularly important in childhood and serves as a powerful preventive tool. On the other hand, obesity and arterial hypertension rates are increasing in children, representing a huge risk for developing major cardiovascular and metabolic diseases in adult life. Of fundamental importance is the modality and volume of exercise required to obtain benefits. In this feasibility study, we considered a group of obese children, studied before and after a 12-week online exercise training program, and subdivided the participants into two groups considering the volume of exercise performed (above or below 1200 MET·min/week). This threshold level was applied in two different ways: subdivision A considered the total weekly physical activity volume (considering both time spent walking for at least 10 min consecutively and time spent performing structured exercise) and subdivision B considered only the weekly volume of structured exercise. We assessed autonomic and metabolic control and auxological and lifestyle parameters. We observed that the improved volume of structured exercise was associated with reduced arterial pressure percentile only in subdivision B and an improvement in markers of vagal and metabolic control was evident. Moreover, the 12-week online exercise training program, defined considering individual fitness level and progressively adapted as the goal was reached, proved to be sustainable from an economical and organizational point of view.

## 1. Introduction

Childhood and youth are ideally the best periods in life to instill healthy behaviors [1], thereby fostering wellbeing and preventing chronic non-communicable diseases (CNCD). On the other hand, overweight, obesity, and the prevalence of hypertension are increasing, particularly in the initial phases of life [2,3], thus determining a dramatic increase in the risk of diseases such as diabetes, coronary artery disease, and cancer [3,4]. Unhealthy lifestyles, such as poor nutrition and sedentariness, contribute to this phenomenon. Additionally, recent data show that obesity [5] and an unhealthy lifestyle, in particular sedentariness [6], are linked to poor prognosis in COVID-19 patients, thus expanding the role of lifestyle in determining health status further to CNCD.

Physical activity is one of the major determinants of a healthy lifestyle, in addition to healthy nutrition, not smoking, good sleep hygiene, and the capability to manage stress [7,8]. Regular physical activity is particularly important in childhood and youth as it grants immediate (improvement of wellbeing, scholastic performance, social relationships [9], etc.), and long-term benefits (prevention/treatment of many diseases), and it may be considered a sustainable tool [10] for the individual and environment. On the other hand, epidemiological data from the USA show that only 28.3% of youth aged 6 to 11 years are active for ≥60 min every day of the week, thus meeting more recent guideline recommendations [3,11,12,13], and this decreases to 16.5% of youth aged 12 to 17 years [4]. European Commission data show that 32.4% of Italian children aged 8–9 years are estimated to meet sufficient physical activity levels (considering WHO guidelines), and this percentage decreases to 11.9% of children aged 11 years old and further decreases to 6.8% of children aged 15 years old [14].

International guidelines [11,12,13] indicate that children and adolescents should perform moderate-to-vigorous intensity, mostly aerobic, physical activity at least an average of 60 min every day of the week and that vigorous-intensity aerobic activities, as well as activities that strengthen muscle and bone, should be incorporated at least 3 days a week. A recent paper [15] reported that a minimum of 20 min/day of vigorous endurance exercise may be best for maximizing cardiorespiratory fitness in adolescence, a parameter that a growing body of epidemiological and clinical evidence [16] considers to be a potentially strong predictor of mortality, showing that the intensity of endurance exercise may be a determinant of the beneficial effect of exercise programs. 

Recent data on the association between walking pace and telomere length [17] (a parameter associated with chronic diseases and proposed as a marker of biological age) underlined the importance of exercise intensity, showing that only brisk walkers had significantly longer telomeres than slow walkers. 

An exercise program, and a lifestyle program in general, may be considered efficacious only if it leads to an improvement of the underlying pathogenetic mechanisms that determine a disease or risk of developing a disease. The mechanisms that are potentially improved by exercise are multifarious and complex, ranging from improved immunological and metabolic profiles to an improvement in hemodynamics [1,4,7,8,11,16,18]. An amelioration in cardiac autonomic regulation (CAR, autonomic control of cardiovascular system) may also play an additional, but little recognized, beneficial role [19], generating a “risk factor gap” above and beyond usual treatments. Several chronic diseases, such as arterial hypertension and diabetes, are characterized in adulthood [20,21] and also in childhood and adolescence [22,23] by autonomic nervous system (ANS) impairment, which may be reversed by exercise training and/or healthy nutrition programs [10,22,24,25,26,27]. 

The literature is particularly rich with scientific data demonstrating that exercise may positively interfere with many mechanisms underlying chronic diseases, rendering it a well-recognized preventive and therapeutic tool [1,4,7,8,11,16,18,22,24,25,26,27,28]. However, the implementation of efficacious exercise programs is less defined [7,8,12,29]. Generally, programs that group children together, such as school-based programs or participation in team sports [12], are considered more effective than home-based exercise programs, since they also foster socialization and play an educational role. On the other hand, these approaches may present some economical and organizational barriers [30,31], such as the need for transportation to the gym or sports field, lack of parental support, cost, and family time management. These barriers may have a particular impact on families of low socio-economical level or in a country, such as Italy, where time spent at school is limited. Moreover, other important barriers, such as lack of skills, motivation, enjoinment, and peer support, as well as feeling shy about physical activity in public [31,32], need to be accounted for in the case of obese children and adolescents. Home-based exercise intervention programs may help in overcoming some of these barriers [33,34,35]. As previously reported [36], remote physical activity programs may provide a valuable strategy for fostering compliance with physical activity guidelines, representing an opportunity for pediatric subjects with obesity to stay healthy. On the other hand, their effectiveness may vary [12,33,37] according to the level of support granted by exercise professionals and the level of personalization of the proposed program.

The goal of this feasibility study was to verify the effectiveness on cardiac autonomic regulation and metabolic parameters of a supervised, home-based exercise program specifically designed to meet obese children’s needs and be enjoyable and sustainable from an economical and organizational point of view in order to facilitate acceptance and compliance. In particular, we assessed the autonomic and metabolic effects considering the volume of physical activity actually performed by children. 

## 2. Materials and Methods

We consecutively enrolled 35 Caucasian children and adolescents (14 females/21 males) admitted to our pediatric outpatient clinic for obesity by their general practitioner or by their primary care pediatric consultant between March 2021 and December 2021. We studied them before (T0) and after (T1) a 12-week online exercise training protocol.

To be included in our study, the children must have been aged between 8 and 13 years, have a body mass index (BMI) z-score ≥ 2 (according to World Health Organization [38]), and have Italian language competency. The exclusion criteria were: known secondary obesity conditions, no comprehension of Italian language, cardiovascular and respiratory chronic diseases, orthopedic problems, and absolute contraindications to practicing physical activity.

This study was approved by the institutional ethics committee (Milano Area 1 protocol number 2020/ST/298, approval date 2 December 2020) and conducted in accordance with the Helsinki Declaration of 1975, as revised in 2008.Written consent was obtained by all participants or their responsible guardians once they were well informed about the study.

### 2.1. Clinical Evaluations

All children underwent the following assessments.

#### 2.1.1. Clinical, Auxological, and Hemodynamic Assessment

In all patients, weight, height, waist circumference (WC), pubertal stage according to Marshall and Tanner [39,40], BMI, waist-to-height ratio (WHtR) [41], triponderal mass index [42], and visceral adiposity index (VAI) [43] were considered as adiposity indexes related to cardiometabolic risk [44]. A basal musculoskeletal assessment was employed in order to exclude the presence of musculoskeletal limitations to the exercise program. Weight was measured with patients not wearing shoes and in light clothing, standing upright in the center of the scale platform (Seca, Hamburg, Germany), facing the recorder, hands at the sides, and looking straight ahead. Standing height was measured using a Harpenden stadiometer (Holtain Ltd., Cross-well, Crymych, UK) with a fixed vertical backboard and adjustable headpiece [44].

WC was performed in the horizontal plane midway between the lowest ribs and iliac crest, using a flexible inch tape [44].

BMI was calculated as body weight (kilograms) divided by height (meters squared) and was transformed into BMI z-scores using WHO reference values [38]. 

Other adiposity indexes were calculated as follows [42,43]:

WHtR = WC/Ht

TMI = weight (kg)/height (m)^3^

VAI

Male = [WC/(39.68  +  (1.88 × BMI))] × (TG/1.03) × (1.31/HDL-C);

Female = [WC/(36.58  +  (1.89 × BMI))] × (TG/0.81) × (1.52/HDL-C)

Pubertal stages according to Tanner were classified as follows: prepubertal stage 1 = Tanner 1; middle puberty stage 2 = Tanner 2–3; and late puberty stage 3 = Tanner 4–5 [39,40]. 

Systolic arterial pressure (SAP) and diastolic arterial pressure (DAP) were measured twice, in the supine position, using an electronic mercury sphygmomanometer (A & D Medical, Tokyo, Japan) with an appropriately sized cuff on the right arm after 5 min of rest [45]; the second measurement was used for analysis. We determined the blood pressure percentile for each child, following recent guidelines [46,47,48]. Moreover, every child underwent a basal electrocardiogram and echocardiogram in order to verify the absence of main cardiac diseases that might contraindicate or limit exercise training, particularly at high intensities.

#### 2.1.2. Metabolic Assessment

A blood sample for the evaluation of fasting blood glucose (FBG), total cholesterol (TOT Cho), high-density lipoprotein (HDL C) cholesterol, triglycerides (TG), and insulin was obtained in fasting state between 8:30 and 9:00 a.m. and analyzed the same morning by standard methods (Advia XPT, Siemens Healthcare). As a surrogate of insulin resistance (IR), we considered the homeostasis model assessment (HOMA-IR) calculated as insulin resistance = (insulin × glucose)/22.5 [49] and the triglyceride and glucose (TyG) index was calculated as ln[fasting triglycerides (mg/dL) × fasting plasma glucose (mg/dL)/2 ]) [50].

#### 2.1.3. Lifestyle Assessment

An ad hoc questionnaire was employed to quantify lifestyle [51,52,53,54,55]:

- Nutrition was assessed using the American Heart Association Healthy Diet Score (AHA score) [8], taking into consideration fruit/vegetables, fish, sweetened beverages, whole grain, and sodium consumption (assessment of the latter was adapted to Italian eating habits) [55].
- The lifestyle questionnaire inquired also about hours of sleep/day, hours of sedentariness/week, and perceptions of quality of sleep, health, and school performance (assessed using evaluation scales from 0 (‘worst quality’) to 10 (‘best quality’) for each measure).
- Physical activity (total activity volume) was assessed by a modified version of the commonly employed short version of the International Physical Activity Questionnaire (IPAQ) [52,53], which focuses on intensity (nominally estimated in metabolic equivalents (METs) according to the type of activity) and duration (in min) of physical activity. We decided to employ this questionnaire, even if it was designed for adults, because it has the advantage of furnishing a numeric parameter of exercise volume (expressed in METs) capable of reflecting the total exercise volume.

We considered the following levels: brisk walking (≈3.3 METs), other activities of moderate intensity (≈4.0 METs), and activities of vigorous intensity (≈8.0 METs).

In accordance with current guidelines [11,12], these levels were used to assess the weekly exercise volume, using the following equations:

▪ (METsTOT) Total weekly physical activity volume [MET·min/week] = (3.3 × minutes of brisk walking × days of brisk walking) + (4.0 × minutes of other moderate intensity activity × days of other moderate intensity activities) + (8.0 × minutes of vigorous intensity activity × days of vigorous intensity activity).
▪ (METsMV) Weekly physical activity volume calculated only considering other activities of moderate intensity and activities of vigorous intensity [MET·min/week] = (4.0 × minutes of other moderate intensity activity × days of other moderate intensity activities) + (8.0 × minutes of vigorous intensity activity × days of vigorous intensity activity). METsMV may be considered as the total weekly volume of structured exercise.

The population of our study was subdivided into two groups: those reaching the physical activity goals suggested by the latest guidelines [11,12], corresponding to at least an average of 60 min/day of moderate-to-vigorous intensity, mostly aerobic, physical activity (above 1200 MET·min/week, and those who did not reach the physical activity goals (below 1200 MET·min/week. Accordingly, we considered the physical activity volume reached at the end of the training.

The threshold level of 1200 MET·min/week was obtained in two different ways:

- Subdivision A considered the total weekly physical activity volume (METsTOT), i.e., considering both time spent walking (at least for 10 min consecutively) and time spent performing structured exercise (other moderate intensity activities and vigorous intensity activities)
- Subdivision B considered only the weekly volume of structured exercise (METsMV) (only other moderate intensity activities and vigorous intensity activities).

The results considering these two different subdivisions are reported respectively in Table 1 and Table 2.

We considered these two different subdivisions in order to unveil the possible different effects associated with structured exercise (generally of higher intensity) or with physical activities of less intensity, such as walking.

All children were equipped with an activity tracker (Fitbit Charge 2 ©, Fitbit Inc, San Francisco, CA, USA) to monitor their heart rate during the day and during the supervised training sessions.

#### 2.1.4. Physical Fitness (PF) Assessment

Prior to starting the training program, all the children were tested for cardiovascular fitness, as follows:

- Six-minute walk test (6MWT). This field test is considered a valid and reliable tool for measuring PF in children and is widespread, has inexpensive equipment, and is easy to administer in a clinical setting [56]. The 6MWT was performed according to international administration guidelines [57]. The children were instructed by the trainers to walk the greatest distance possible while maintaining their own pace. Standardized encouragement and information about the remaining time were given to the children every minute; for example, “you are doing well” or “keep up the good work” [58]. Children were permitted to stop (if required) during the test but were instructed to resume walking once able and the covered distance was registered in meters. Test-retest reliability was undertaken, and the intraclass correlation coefficient (95% confidence interval) was calculated as 0.94 (0.89–0.96).
- After an adequate recovery time, children were interviewed by the same investigator to assess perceived physical fitness and physical activity level, respectively, using the International Fitness Enjoyment Scale (IFIS) and Physical Activity Questionnaire for Older Children (PAQ-C) questionnaires.
- The International Fitness Enjoyment Scale (IFIS) questionnaire is a self-reported, easy, and rapid fitness scale previously validated in several European countries and languages. It describes physical fitness as an indicator of physical competence [59]. The IFIS is composed of a 5-point Likert scale (from 1 ‘very poor’ to 5 ‘very good’), with questions focused on five areas of fitness: general fitness, cardiorespiratory, strength, speed-agility, and flexibility. The IFIS has high validity and moderate-to-good reliability (average weighted Kappa: 0.70 and 0.59) for school-aged children.
- The Physical Activity Questionnaire for Older Children (PAQ-C) evaluates the weekly amount of physical activity reported by children. This questionnaire was verified to be adequate for school-aged children (approximate ages between 8 and 14). The PAQ-C is recognized as a valid and reliable measurement of general physical activity level from childhood to adolescence. The PAQ-C utilizes cues such as break time at school and evening physical activity to ameliorate the recall ability of children. The PAQ-C is cost- and time-efficient, simple to administer, and displays normal distribution properties. The PAQ-C is shown to have good reliability and an intraclass correlation (ICC) = 0.96 [60].

#### 2.1.5. Cardiac Autonomic Regulation (CAR) Assessment

On the day of autonomic evaluation, all subjects arrived at the clinic avoiding caffeinated beverages since awakening as well as heavy physical exercise in the preceding 24 h. Recordings were performed between 04:00 am and 06:00 pm in an air conditioned, quiet room. After a preliminary 10-min rest period in the supine position, electrocardiogram (ECG) and respiratory activity (piezoelectric belt) were continuously recorded over a minimum 5-min period using a two-way radiotelemetry system (Marazza, Monza Italy). Subsequently, subjects were asked to stand up unaided and remain in the upright position for 5 min, during which recordings were maintained. Data were acquired with a PC at 250 samples/second using a custom built software tool (HeartScope) that automatically provided a series of indices describing heart rate variability (HRV) in the time domain: RR interval (in msec) and RR interval variability (RRTP) (assessed as total power, i.e., variance, in msec^2^), taken as simple classifiers typical of vagal control [21,61,62]. A series of indices were also provided in the frequency domain: autoregressive spectral components both in the low frequency (LF, center frequency ≈ 0.1 Hz) and high frequency (HF, centered with respiration, ≈0.25 Hz) (assessed in msec^2^ as well as in normalized units [nu]) and markers of prevalent sympathetic and vagal activities, indicating sinoatrial node function [21,62].

Based on all of these assessments before starting the training, a medical exercise prescription defining the modality, intensity, duration, frequency, and progression of exercise was created for each participant, who were individually followed by exercise physiologists using a Zoom online platform.

### 2.2. Exercise Training Protocol

Children performed a 12-week online training program supervised by two sport specialists using the Zoom online platform (California, USA). The training protocol consisted of three 60-min sessions per week, over 12 weeks, for a total of 36 sessions. Each session was streamed in real-time using the Zoom platform, which allowed live interaction among the instructors and children. Two sport specialists supervised every training session, which usually consisted of different typology and intensity of exercises, defined considering individual fitness levels and progressively adapted as each individual reached the goal. In detail, all sessions were divided into three sub-sessions: an initial warm-up of approximately 5 min to ensure the correct preparation of the children for the subsequent exercises, the main training that consisted of a combination of aerobic and muscular routines for approximately 50 min, and a final part of cool down of approximately 5 min to ensure the return of the body to the rest condition. The exercise physiologist recorded that the training session was actually performed by the child. All the proposed exercises were playful and recreative activities; for example, we proposed motor and active fabulation, dancing to music, imitation games, circuit training in a playful way, playing with small objects such as balls, books, pencils, and paper, and paths through the furniture. The activities started from low intensity and progressed through the training to moderate and high intensities with values of maximum heart rate ranging from 50% to 80%, as calculated using the Tanaka equation [63] after clinical verification that no cardiovascular contraindications were present for the execution of vigorous exercise. Shorter bouts of yoga and mindfulness were proposed as cool-down activities [64]. The main goal of the intervention was to administer a simple and enjoyable online training sessions with interaction and adaptable loads that were affordable and sustainable for families and had no cost or transportation difficulties. Starting 30 min from the beginning of the session, the trainers acquired the children’s heart rates in order to control the intensity of the training using the Fitbit Charge 2 © (Fitbit Inc, San Francisco, CA, USA).

**Figure 1:**
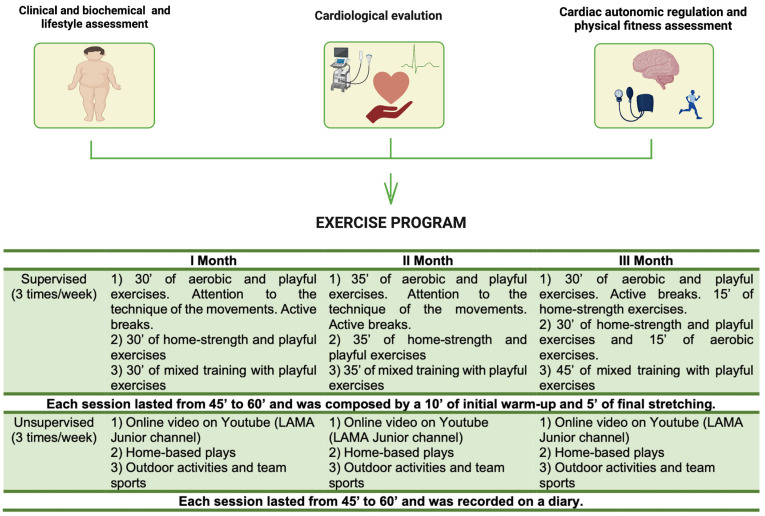
Exercise program.

To better comply with the guidelines [11,12] recommending 60 min of moderate-to-vigorous exercise per day for children and adolescents, we provided a dedicated YouTube channel “LAMA Junior” with adapted exercise routines for days without supervised training [34]. See Figure 1.

Throughout the exercise program, patients were asked to maintain a healthy diet but no standard modifications were made.

### 2.3. Statistical Analysis

Data are presented as the median (25–75 percentile). The significance of differences was assessed with repeated measures GLM (General Linear Model), considering group and intervention as factors. Differences in pubertal stage between T0 and T1 were assessed using the Chi-squared test. Bivariate Pearson’s correlation analysis was employed with two-tailed significance.

Computations were performed using IBM SPSS Statistics for Windows, version 27 (IBM Corp., Armonk, NY, USA) with a PC (DELL). A value of *p* < 0.05 was taken as the threshold for statistical significance.

## 3. Results

Throughout the program period, no significant changes in pubertal stage were noted. (Stage 1 = 19 vs. 17, Stage 2 = 6 vs. 8, Stage 3 = 10 vs. 10, *p* = 0.82).

As specified in the methods, we subdivided the population into two groups: those reaching the physical activity goals suggested by the latest guidelines (corresponding to at least an average of 60 min/day of moderate-to-vigorous intensity, mostly aerobic, physical activity, i.e., above 1200 MET·min/week) and those who did not reach the physical activity goals (i.e., below 1200 MET·min/week). 

Table 1 reports the results of subdivision A considering the total weekly physical activity volume (METsTOT) (i.e., both time spent walking and time spent performing structured exercise), while Table 2 reports the results of subdivision B considering only the weekly volume of structured exercise (METsMV). The tables report data before (T0) and after (T1) exercise intervention.

### 3.1. Clinical, Auxological, Hemodynamical and Metabolic Data

Subdivision A showed that significant differences were present between conditions in clinical auxological parameters (BMI z-score *p* = 0.004 and TMI *p* < 0.001) and between groups in metabolic parameters (total cholesterol *p* = 0.048). No significant differences were noted regarding systolic arterial pressure, diastolic arterial pressure percentile, and heart rate. Similarly, there was no difference in autonomic parameters for RRV.

Subdivision B showed significant differences between conditions in clinical auxological parameters (BMI z-score *p* = 0.033, TMI *p* = 0.003, and VAI *p* = 0.012) and metabolic parameters (total cholesterol *p* = 0.048), with an interaction for HOMA-IR (*p* = 0.05) and VAI (*p* = 0.048). No significant differences were noted regarding systolic arterial pressure, diastolic arterial pressure percentile, and heart rate. Borderline significance was noted in RR interval (*p* = 0.062).

### 3.2. Lifestyle Data

No significant differences were noted in subdivisions A and B regarding hours sleep/day and school performance. Quality of sleep and health perceptions were slightly different between groups in subdivision A. Hours of sedentariness/week was slightly different between groups in subdivisions A and B. The AHA score (quality of nutrition index) was slightly different between groups only in subdivision B, and it improved after training. As expected, the volume of performed physical activity was significantly increased after intervention in both subdivisions A and B (*p* = 0.008 and *p* < 0.001, respectively). Interestingly, in both subdivisions, the subjects who performed higher volumes of physical activity after intervention were also characterized by higher volumes of physical activity before intervention (*p* < 0.001 and *p* = 0.012, respectively), with significant interaction (*p* = 0.030 and *p* = 0.012, respectively), suggesting that the increase was significantly higher in group 2 for both subdivisions.

### 3.3. Physical Fitness Assessment Data

The covered distance of 6MWT and PAQ-C score were significantly increased after intervention in groups 1 and 2, for both subdivisions A and B. No significant differences between groups or conditions were observed in the IFIS score for both subdivisions A and B. 

### 3.4. Cardiac Autonomic Regulation Data

No significant differences between groups or condition were observed in any of the autonomic variables in subdivision A. 

Subdivision B unveiled an interaction effect in RRHFnu (marker of prevalent vagal modulation of the sinoatrial node) and LF/HF ratio, being slightly increased only in those subjects who performed higher volumes of structured exercise.

Table 3 reports the Pearson’s correlations among changes (Δ) between T0 and T1 for the major variables. Notably, ΔMETsTOT was correlated, as expected, with ΔRRTP (change in total variance of heart rate variability), while ΔMETsMV was significantly correlated not only with ΔRRTP but also with ΔRR (change in RR interval) and ΔHFnu. Moreover, ΔMETsMV was significantly correlated (r = −0.400, *p* = 0.017) with Δ SAPpc (change in percentile of systolic arterial pressure), suggesting that only structured exercise was associated with a reduction of the percentile of SAP. Additionally, ΔRRTP and ΔRR were significantly correlated with ΔSAPpc.

## 4. Discussion

In this feasibility study, we observed that only the improvement in the volume of structured exercise was associated with the reduction in arterial pressure percentile and improvement of markers of vagal control in obese children. Moreover, structured exercise improved the auxological and metabolic parameters. The increase in volume of structured exercise was obtained through a 12-week exercise training protocol delivered online by exercise physiologists [34,54], using an individualized exercise prescription that was progressively adapted as each individual reached their goal and was specifically designed to meet the needs of obese children and their families, while being economically and organizationally sustainable.

The beneficial effects of exercise are well reported in the literature and exercise is now considered a mandatory preventive and therapeutic strategy in all CNCD, particularly in youth [11,12,13], as underlined in the introduction. Of fundamental importance is the dose/volume of physical activity required to obtain such benefits. While international guidelines [11,12,13] clearly indicate the volume of required physical activity and the need to perform moderate and vigorous exercise at a young age, their practical translation into everyday life is sometimes less specific. Simply walking or practicing sports, without paying attention to the volume and particularly the intensity of exercise, is considered efficacious. This phenomenon is well emblemized by the importance given to the number of steps walked every day [65] independent of the speed of walking. On the contrary, the literature shows that the benefits of endurance exercise are also linked to the level of cardiorespiratory fitness [15,16] in children and that only brisk walkers had significantly longer telomeres than slow walkers among adults [17], thereby showing a direct correlation between exercise intensity and longevity. In this paper, we observed that only the volume of exercise due to structured moderate-to-vigorous intensity exercise was associated with improved cardiac autonomic control, reduced systolic arterial pressure, and improved auxologic and metabolic parameters. Of particular interest is the reduction in the HOMA-IR index, which suggested reduced insulin resistance after training, confirming the relevant influence of different types of exercise on insulin resistance [66]. In contrast, we observed only a slight change in the auxologic parameters, such as BMI z-score and TMI, considering that the MET count also included METs linked to simple walking for more than 10 min.

The data in our study, albeit preliminary, suggest that only structured exercise was capable of inducing an improvement in autonomic and metabolic control. Of particular interest is the observation that the change in the SAP percentile with training was associated with the change in structured exercise volume and changes in ANS variable markers of vagal control. Arterial hypertension is a condition that characterizes childhood obesity [2,3,6] and increases the risk of major cardiovascular disease over the lifespan. Many studies have demonstrated that exercise is a powerful tool to prevent/treat arterial hypertension and obesity [11,12,13]. The data in the current study corroborate previous observations in which improved autonomic cardiac regulation was among the benefits of exercise in the management of hypertension and obesity [10,22,24,25,26,27]. 

Another interesting point to discuss is the methodology employed to help children with obesity become physically active. Participation in team sports or programs that generally group children together are considered more effective, but they may present some practical barriers (economical, organizational, and psychological), as reported in the introduction. In this paper, we observed that a home-based, online exercise training program could overcome, albeit in part, these barriers, and could be enjoyable and sustainable from an economical and organizational point of view, thus facilitating acceptance and compliance of children and their families. This observation was corroborated by 86% of children in our study remaining in the program after completing the final evaluations.

In addition, the possibility of starting exercise at home, thus avoiding interaction with peers while improving their physical performance, may result in an improvement of self-efficacy [67] and, consequently, motivate obese children to exercise in a different environment close to their peers. A fundamental characteristic of the employed protocol [34] was the balance between the possibility of being delivered online and tailoring the proposed modality/intensity of exercises to the child’s needs, preferences, and reached goals [54]. An individualized clinical assessment and exercise prescription was essential to the achievement of this goal, which required exercise physiologists to interact online with children, design enjoyable exercises, and adjust the program during the training period. In contrast [12,37,68], home-base exercise programs that were not tailored to individual needs showed poor or no efficacy in previous studies.

In this observational study, we also observed that children with obesity who significantly increased their exercise volume through the intervention program were already characterized by higher exercise volumes and less sedentary hours (considering both subdivisions) before starting the intervention program. This finding suggests that other factors, in addition to the methodology employed in the exercise program, need to be considered, such as psychological and social conditions [9,67]. Although we do not have data to support this hypothesis, we observed that the group of children who actually increased their exercise volume in subdivision A were characterized before the intervention by slightly higher health and quality of sleep perceptions.

Throughout the exercise program, children with obesity were asked to maintain a healthy diet but no standard modifications were made. Nevertheless, the group of subjects who significantly increased the volume of structured exercise was also characterized by a higher AHA score, suggesting an improvement in the quality of nutrition. Further supporting the hypothesis expressed in the previous paragraph, we also observed that this group was characterized by better AHA scores before the intervention.

Some limitations of this study must be acknowledged. First, this study was an observational feasibility study and the group size was small, thus limiting the interpretative value of the results. Additionally, we did not consider a control group (subjects studied before and after sham intervention). Nevertheless, we observed in other studies that sham interventions did not induce modifications of the autonomic nervous system per se [69,70], corroborating the idea that the observed changes were attributable to functional remodeling. Second, only 19 children (54.29%) wore the activity tracker both during the day and during the training sessions and were considered for the specific analysis. Interestingly, we observed a significant correlation (r = 0.445, *p* = 0.049) between the total steps recorded by the tracker and the total volume of structured exercise (METsMV), while no significant correlation (r = 0.235, *p* = 0.3.19) was observed with the total volume of physical activity (METsTOT). Third, the children’s diets were not standardized, allowing for a potential bias. Moreover, we did not have data regarding immunological control (another main control system that plays an important role in cardiometabolic conditions) and psychological and social aspects. These are important factors to consider when studying the effects of intervention programs in subjects with obesity. In addition, the intervention lasted only 12 weeks and we do not have data regarding the long-lasting effects of our model. Therefore, in view of the small number of subjects and overall feasibility of the design, generalizing the results is not possible.

## 5. Conclusions

In conclusion, this study showed the feasibility of an online exercise program as a practical tool to improve cardiac autonomic control and hemodynamic, metabolic, and auxological parameters in a group of children with obesity, rendering it a valuable strategy for fostering compliance with physical activity guidelines and providing an opportunity for pediatric subjects with obesity to stay healthy, thus managing the increased risk of CNCD. Moreover, by guaranteeing that an adequate level of exercise intensity was reached, this study underlines the importance of structured exercise in obtaining significant health benefits. The benefits of exercise overcome those of “simple” weight reduction (which in this study was present when considering subdivision A), which are also linked to improvements in important endocrine and autonomic control mechanisms (which in this study was evident when considering subdivision B). In addition, the ability of exercise to affect control mechanisms, particularly cardiac autonomic control, seems only to be evident if exercise reaches an adequate intensity, i.e., even at the same exercise volume (1200 METs), the effect may differ depending on the intensity of the exercise performed. With this in mind, we have to consider that this was a feasibility study. Therefore, the capability of the exercise program to affect CAR and its economical/organizational sustainability must be verified. On the basis of the present observations, larger and longer lasting studies aimed at confirming these preliminary satisfactory data seem justified.

## Figures and Tables

**Table 1:** Summary of descriptive data of the study population subdivided into two groups considering total volume of physical activity (METsTOT) (Subdivision A).

Indices	Groups		Significance		
	Group 1 n = 12	Group 2 n = 23			
	Below 1200 METs	Above 1200 METs	Between Groups	Between T0–T1	Interaction
	Median (Percentile 25°; 75°)	Median (Percentile 25°; 75°)			
HR T0 [b/min]	90.76 (83.38; 97.48)	82.15 (75.67; 85.90)	0.283	0.843	0.163
HR T1	90.80 (78.83; 95.34)	83.52 (77.86; 95.90)			
RR T0 [msec]	661.20 (615.73; 719.99)	730.38 (698.48; 792.92)	0.186	0.898	0.289
RR T1	660.93 (629.34; 761.20)	718.41 (625.64; 770.63)			
RRTP T0 [msec^2^]	2015.38 (700.66; 4175.22)	2070.68 (1174.64; 4358.51)	0.726	0.694	0.604
RRTP T1	1941.73 (1036.30; 2996.04)	1974.04 (880.01; 3978.67)			
RRLFa T0 [msec^2^]	278.57 (152.21; 1536.34)	569.95 (324.55; 1064.11)	0.613	0.911	0.795
RRLFa T1	414.05 (246.20; 1126.03)	679.74 (218.92; 1833.57)			
RRHFa T0 [msec^2^]	565.22 (124.40; 1610.27)	600.91 (198.91; 1718.47)	0.917	0.423	0.331
RRHFa T1	461.04 (252.52; 1177.21)	630.36 (235.87; 1128.62)			
RRLFnu T0 [nu]	30.66 (25.04; 48.65)	46.38 (31.23; 55.80)	0.267	0.264	0.727
RRLFnu T1	41.86 (34.10; 44.14)	41.52 (33.52; 65.68)			
RRHFnu T0 [nu]	52.41 (37.54; 61.06)	46.00 (28.39; 59.02)	0.808	0.310	0.367
RRHFnu T1	47.47 (32.09; 56.59)	43.79 (28.31; 57.69)			
RRLF/HF T0 [.]	0.58 (0.41; 1.30)	1.01 (0.46; 2.27)	0.299	0.660	0.691
RRLF/HF T1	0.91 (0.55; 1.46)	0.95 (0.59; 2.32)			
SAPpc T0 [%]	78.00 (35.50; 87.00)	80.00 (63.00; 92.00)	0.058	0.464	0.895
SAPpc T1	58.50 (49.50; 65.00)	80.00 (51.00; 93.00)			
DAPpc T0 [%]	75.00 (56.00; 92.00)	71.00 (59.00; 95.00)	0.348	0.376	0.068
DAPpc T1	61.50 (42.50; 84.50)	78.00 (59.00; 91.00)			
AHA score T0 [.]	1.50 (1.00; 2.50)	2.00 (1.00; 3.00)	0.157	0.180	0.180
AHA score T1	2.00 (1.00; 2.50)	3.00 (2.00; 3.00)			
Hours of sleep T0 [h/day]	8.00 (8.00; 9.00)	8.50 (8.00; 9.00)	0.156	0.208	0.402
Hours of sleep T1	9.00 (7.00; 9.00)	9.00 (8.00; 9.00)			
Quality of Sleep T0 [.]	8.50 (6.00; 10.00)	10.00 (8.00; 10.00)	**0.020**	0.351	0.829
Quality of SleepT1	8.50 (4.50; 9.50)	9.00 (9.00; 10.00)			
Health T0 [.]	7.00 (6.00; 9.00)	8.00 (6.00; 10.00)	**0.051**	0.215	0.215
Health T1	6.50 (5.00; 9.00)	8.00 (6.00; 9.00)			
School Performance T0 [.]	8.00 (7.00; 10.00)	9.00 (7.00; 10.00)	0.450	0.215	0.907
School Performance T1	8.50 (7.00; 9.00)	8.00 (7.00; 9.00)			
Sedentariness T0 [h/week]	68.00 (61.00; 82;00)	56.00 (49.00; 68.00)	**0.038**	0.229	0.775
Sedentariness T1	66.50 (52.00; 84.50)	61.00 (28; 68.00)			
METsMV T0 [MET·min/week]	240.00 (0.00; 880.00)	480.00 (0.00; 720.00)	0.056	0.119	0.117
METsMV T1	480.00 (330.00; 670.00)	960.00 (720.00; 1800.00)			
METsTOT T0 [MET·min/week]	361.50 (153.00; 966.25)	918.00 (495.00; 1635.00)	**0.000**	**0.008**	**0.030**
METsTOT T1	752.25 (538.50; 960.50)	1860.00 (1395.00; 2580.00)			
BMI z-score T0 [.]	2.13 (1.65; 2.52)	2.04 (1.84; 2.41)	0.678	**0.004**	0.200
BMI z-score T1	2.00 (1.35; 2.54)	1.97 (1.82; 2.49)			
WHtR T0 [.]	0.59 (0.57; 0.62)	0.59 (0.57; 0.64)	0.702	0.223	0.142
WHtR T1	0.58 (0.54; 0.60)	0.58 (0.55; 0.60)			
FBG T0 [mg/dL]	89.50 (87.00; 91.50)	87.00 (85.00; 95.00)	0.638	0.683	0.406
FBG T1	93.00 (86.50; 99.00)	89.00 (85.00; 95.00)			
Insulin T0 [mg/dL]	17.55 (11.25; 25.85)	17.40 (13.14; 30.70)	0.343	0.515	0.375
Insulin T1	15.40 (8.80; 30.75)	18.00 (12.00; 30.10)			
HOMA-IR T0 [.]	3.97 (2.50; 5.59)	3.87 (2.76; 6.64)	0.354	0.596	0.363
HOMA-IR T1	3.78 (1.86; 7.02)	3.78 (2.77; 5.72)			
TG T0 [mg/dL]	93.00 (64.50; 117.50)	113.00 (73.00; 150.00)	0.281	0.886	0.331
TG T1	91.00 (60.00; 119.00)	110.00 (65.00; 148.00)			
TOT Chol T0 [mg/dL]	149.00 (123.00; 156.00)	170.00 (150.00; 190.00)	**0.048**	0.314	0.068
TOT Chol T1	143.00 (129.00; 183.00)	167.00 (144.00; 172.00)			
HDL C T0 [mg/dL]	47.00 (37.00; 53.50)	45.00 (41.00; 50.00)	0.990	0.427	0.567
HDL C T1	49.00 (43.00; 52.00)	47.00 (40.00; 50.00)			
TMI T0 [.]	19.13 (18.17; 21.30)	18.64 (17.24; 20.53)	0.637	**0.000**	0.160
TMI T1	18.62 (17.47; 20.91)	18.32 (16.72; 19.68)			
VAI T0 [.]	3.33 (1.60; 4.90)	3.30 (1.58; 4.96)	0.330	0.137	0.392
VAI T1	3.01 (2.01; 4.53)	4.46 (2.48; 5.58)			
TyG T0 [.]	8.26 (8.01; 8.54)	8.56 (7.90; 8.82)	0.523	0.722	0.306
TyG T1	8.35 (7.94; 8.47)	8.47 (7.93; 8.80)			
6MWT T0 [m]	464.00 (427.00; 540.00)	472.00 (438.00; 504.00)	0.596	**0.000**	0.122
6MWT T1	516.00 (482.00. 560.00)	540.00 (500.00; 574.00)			
PAQ-C score T0 [.]	1.92 (1.74; 2.17)	1.97 (1.57; 2.30)	0.840	**0.043**	0.858
PAQ-C score T1	2.39 (1.90; 2.53)	2.25 (1.77; 2.69)			
IFIS score T0 [.]	3.40 (3.20; 4.20)	3.00 (2.80; 3.80)	0.159	0.543	0.653
IFIS score T1	3.80 (3.20; 4.00)	3.40 (2.80; 4.00)			

Abbreviations: T0 = before intervention; T1 = after intervention; HR = heart rate; RR= RR interval; RRTP = RR total power (RR interval variance); LF = low frequency component of RR variability; HF = high frequency component of RR variability; nu = normalized unit; LF/HF = RRLF on RRHF ratio; SAPpc = percentile of systolic arterial pressure; DAPpc = percentile of diastolic arterial pressure; AHA score = American Heart Association Nutrition Score; METsTOT = total weekly physical activity volume; METsMV = weekly physical activity volume calculated only considering other activities of moderate intensity and activities of vigorous intensity volume (i.e., volume of structured exercise); BMI= body mass index; WHtR = waist-to-height ratio; FBG = fasting blood glucose; HOMA-IR = homeostasis model assessment—insulin resistance; TG = triglycerides; TOT Chol = total cholesterol; HDL C = HDL cholesterol; TMI = triponderal mass index; VAI = visceral adiposity index; TyG = triglyceride and glucose index; 6MWT = 6-min walk test; PAQ-C = physical activity questionnaire—children; IFIS = international fitness scale; [.] = arbitrary units. Significant values are evidenced in bold.

**Table 2:** Summary of descriptive data of the study population subdivided into two groups considering weekly volume of structured exercise (METsMV) (Subdivision B).

Indices	Groups		Significance		
	Below 1200 METs	Above 1200 METs	Between Groups	Between T0–T1	Interaction
	Median (Percentile 25°; 75°)	Median (Percentile 25°; 75°)			
HR T0 [b/min]	86.61 (81.86; 95.46)	77.07 (73.44; 83.37)	0.139	0.555	0.344
HR T1	89.55 (80.08; 95.83)	80.87 (68.25; 94.11)			
RR T0 [msec]	692.84 (628.57; 732.96)	778.47 (719.76; 816.99)	0.062	0.721	0.675
RR T1	670.07 (626.13; 749.24)	741.94 (637.58; 879.14)			
RRTP T0 [msec^2^]	1602.01 (775.36; 3131.09)	3215.74 (2070.68; 4532.33)	0.686	0.996	0.552
RRTP T1	1711.46 (1036.30; 2996.04)	2342.19 (815.39; 4338.19)			
RRLFa.T0 [msec^2^]	392.29 (165.69; 1052.10)	952.41 (465.37; 1154.68)	0.995	0.740	0.702
RRLFa T1	498.63 (246.86; 1390.14)	679.74 (138.87; 1833.57)			
RRHFa. T0 [msec^2^]	551.61 (158.16; 1360.03)	773.98 (269.76; 2289.90)	0.754	0.942	0.266
RRHFa T1	474.34 (252.52; 1092.48)	777.43 (235.87; 1485.89)			
RRLFnu T0 [nu]	44.24 (27.10; 51.30)	48.20 (26.04; 69.70)	0.947	0.735	0.063
RRLFnu.T1	42.13 (34.23; 57.47)	34.69 (22.89; 67.94)			
RRHFnu T0 [nu]	49.16 (40.03; 59.66)	49.43 (20.20; 67.22)	0.651	0.952	**0.032**
RRHFnu. T1	42.55 (32.09; 55.36)	55.05 (27.20; 70.76)			
RRLF/HF T0 [.]	0.87 (0.48; 1.29)	0.98 (0.44; 3.53)	0.450	0.546	**0.015**
RRLF/HF T1	1.09 (0.59; 1.68)	0.60 (0.32; 2.58)			
SAPpc T0 [%]	77.00 (46.00; 87)	86.00 (65.00; 94.00)	0.315	0.243	0.197
SAPpc T1	64.5 (56.00; 91.00)	83.00 (50.00; 92.00)			
DAPpc T0 [%]	75.00 (60.00; 92.00)	65.00 (58.00; 95.00)	0.987	0.864	0.768
DAPpc T1	79.5 (49.5; 90.5)	76.00 (55.00; 89.00)			
AHA score T0 [.]	1.00 (1.00; 2.00)	2.00 (2.00; 3.00)	**0.019**	**0.045**	0.359
AHA score T1	2.00 (1.00; 3.00)	3.00 (2.00; 4.00)			
Hours of sleep T0 [h]	8.5 (8.00; 9.00)	8.00 (8.00; 9.00)	0.507	0.575	0.141
Hours of sleep.T1	8.00 (7.50; 9.00)	9.00 (8.00; 9.00)			
Quality of Sleep T0 [.]	9.00 (8.00; 10.00)	10.00 (8.00;1 0.00)	0.233	0.414	0.935
Quality of Sleep T1	9.00 (7.00; 10.00)	9.00 (9.00; 10.00)			
Health T0 [.]	8.00 (6.00; 10.00)	7.00 (6.00; 9.00)	0.744	0.703	0.229
Health T1	8.00 (5.50; 9.00)	7.00 (6.00; 9.00)			
School Performance T0 [.]	9.00 (7.00; 10.00)	8.00 (7.00; 10.00)	0.921	0.160	0.560
School Performance T1	8.00 (7.00; 9.00)	8.00 (6.00; 9.00)			
Sedentariness T0 [h/week]	66.00 (56.00; 78.50)	53.00 (48.00; 56.00)	**0.010**	0.162	0.708
Sedentariness T1	63.00 (52.00; 79.50)	54.00 (26.00; 67.00)			
METsMV T0 [MET·min/week]	240.00 (0.00; 480.00)	720.00 (0.00;1 680.00)	**0.000**	**0.002**	**0.007**
METsMV T1	600.00 (480.00; 720.00)	1800 (1200.00; 2080.00)			
METsTOT T0 [MET·min/week]	648.75 (268.50; 1333.00)	819.00 (0.00; 1920.00)	**0.012**	**0.000**	**0.012**
METsTOT T1	1207.50 (752.25; 1644.00)	2493.00 (1860.00; 2773.00)			
BMI z-score T0 [.]	2.11 (1.91; 2.38)	1.99 (1.69; 2.55)	0.885	**0.033**	0.282
BMI z-score T1	1.99 (1.63; 2.35)	1.90 (1.58; 2.52)			
WHtR T0 [.]	0.59 (0.57; 0.63)	0.59 (0.56; 0.64)	0.647	0.848	0.142
WHtR T1	0.57 (0.56; 0.60)	0.59 (0.55; 0.63)			
FBG T0 [mg/dL]	89.00 (86.00; 92.50)	87.00 (86.00; 96.00)	0.708	0.939	0.594
FBG T1	90.00 (85.50; 96.00)	88.00 (87.00; 96.00)			
Insulin T0 [mg/dL]	16.80 (11.75; 23.80)	18.00 (13.14; 35.00)	0.420	0.088	0.061
Insulin T1	16.00 (10.24; 30.15)	18.20 (14.80; 30.10)			
HOMA-IR T0 [.]	3.80 (2.51; 5.36)	3.87 (2.76; 7.78)	0.372	0.103	**0.053**
HOMA-IR T1	3.75 (2.22; 6.46)	4.27 (3.22; 5.72)			
TG T0 [mg/dL]	96.00 (69.50; 122.50)	121.00 (62.00; 169.00)	0.362	0.876	0.991
TG T1	91.00 (63.00; 120.00)	112.00 (75.00; 156.00)			
TOT Chol T0 [mg/dL]	156.00 (139.00; 187.00)	165.00 (150.00; 190.00)	0.782	**0.048**	0.199
TOT Chol T1	155.50 (133.00; 176.00)	151.00 (144.00; 168.00)			
HDL C T0 [mg/dL]	47.50 (40.50; 55.50)	43.00 (39.00; 48.00)	0.390	0.445	0.675
HDL C T1	48.00 (43.00; 52.00)	46.00 (39.00; 48.00)			
TMI T0 [.]	19.08 (18.30; 21.03)	17.58 (17.15; 19.93)	0.285	**0.003**	0.760
TMI T1	19.07 (17.69; 20.91)	17.48 (16.38; 19.68)			
VAI T0 [.]	3.26 (1.79; 4.85)	3.42 (1.51; 5.53)	0.611	**0.012**	**0.048**
VAI T1	3.61 (2.07; 4.73)	5.34 (2.65; 5.85)			
TyG T0 [.]	8.35 (7.99; 8.66)	8.58 (7.88; 8.97)	0.454	0.998	0.938
TyG T1	8.35 (7.92; 8.62)	8.61 (8.09; 8.84)			
6MWT T0 [m]	464.00 (432.00; 509.00)	490.00 (440.00; 509.00)	0.532	**0.000**	0.263
6MWT T1	520.00 (492.00; 560.00)	564.00 (520.00; 580.00)			
PAQ-C score T0 [.]	1.97 (1.73; 2.17)	1.84 (1.57; 2.39)	0.895	**0.046**	0.731
PAQ-C score T1	2.30 (1.77; 2.53)	2.25 (1.81; 2.69)			
IFIS score T0 [.]	3.00 (2.80; 3.60)	3.20 (3.00; 4.20)	0.693	0.578	0.214
IFIS score T1	3.60 (3.20; 4.00)	3.40 (2.80; 4.00)			

Abbreviations: T0 = before intervention; T1 = after intervention; HR = heart rate; RR = RR interval; RRTP = RR total power (RR interval variance); LF = low frequency component of RR variability; HF = high frequency component of RR variability; nu = normalized unit; LF/HF = RRLF on RRHF ratio; SAPpc = percentile of systolic arterial pressure; DAPpc = percentile of diastolic arterial pressure; AHA score = American Heart Association Nutrition Score; METsTOT = total weekly physical activity volume; METsMV = weekly physical activity volume calculated only considering other activities of moderate intensity and activities of vigorous intensity volume (i.e., volume of structured exercise); BMI= body mass index; WHtR = waist-to-height ratio; FBG = fasting blood glucose; HOMA-IR = homeostasis model assessment—insulin resistance; TG = triglycerides; TOT Chol = total cholesterol; HDL C = HDL cholesterol; TMI = triponderal mass index; VAI = visceral adiposity index; TyG = triglyceride and glucose index; 6MWT = 6-min walk test; PAQ-C = physical activity questionnaire—children; IFIS = international fitness scale; [.] = arbitrary units. Significant values are evidenced in bold.

**Table 3:** Pearson’s correlations among changes between T0 and T1 of main variables.

Δ METsMV	0.844 **							
	0.000							
Δ RR	0.227	0.423 *						
	0.189	**0.011**						
Δ RRTP	0.405 *	0.509 **	0.686 **					
	**0.016**	**0.002**	**0.000**					
Δ RRLFnu	−0.117	−0.204	−0.086	−0.012				
	0.504	0.239	0.622	0.946				
Δ RRHFnu	0.216	0.335 *	0.061	0.088	−0.872 **			
	0.213	**0.049**	0.729	0.617	**0.000**			
Δ LF/HF	−0.137	−0.238	−0.004	0.069	0.721 **	0.716 **		
	0.433	0.168	0.983	0.692	**0.000**	**0.000**		
Δ SAPpc	−0.136	−0.400 *	−0.512 **	−0.368 *	0.214	−0.187	0.061	
	0.436	**0.017**	**0.002**	**0.030**	0.217	0.281	0.729	
Δ BMI z-score	−0.088	−0.008	−0.189	−0.243	0.099	−0.103	−0.079	0.094
	0.617	0.963	0.278	0.160	0.572	0.555	0.654	0.590
	Δ METsTOT	Δ METsMV	Δ RR	Δ RRTP	Δ RRLFnu	Δ RRHFnu	Δ LF/HF	Δ SAPpc

Abbreviations: Δ = change between T0 and T1; METsTOT = total weekly physical activity volume; METsMV = weekly physical activity volume calculated only considering other activities of moderate intensity and activities of vigorous intensity volume (i.e., volume of structured exercise); RR = RR interval; RRTP = RR total power (RR interval variance); LF = low frequency component of RR variability; HF = high frequency component of RR variability; nu= normalized unit; LF/HF= RRLF on RRHF ratio; SAPpc= percentile of systolic arterial pressure. BMI= body mass index. Significant values are evidenced in bold. **: correlation is significant at the 0.01 level (2-tailed). *: correlation is significant at the 0.05 level (2-tailed).

## Data Availability

Data will be available on justified request. We have not yet uploaded the data because they are part of an ongoing study on the effects of exercise in children and other papers may be prepared using them.
